# Phosphoproteomic changes in response to anoxia are tissue-specific in the anoxia-tolerant crucian carp (*Carassius carassius*)

**DOI:** 10.3389/fphys.2024.1407834

**Published:** 2024-05-30

**Authors:** Anette Johansen, Bernd Thiede, Jan Haug Anonsen, Göran E. Nilsson

**Affiliations:** ^1^ Department of Biosciences, University of Oslo, Oslo, Norway; ^2^ Norwegian Research Centre AS, Climate and Environment Department, Stavanger, Norway

**Keywords:** anoxia, reoxygenation, crucian carp, brain, liver, mass spectrometry, posttranslational modifications

## Abstract

Crucian carp (*Carassius carassius*), a freshwater fish, can survive chronic anoxia for several months at low temperatures. Consequently, anoxia-related physiological and biochemical adaptations in this species have been studied for more than half a century. Still, despite for the well-known role of protein phosphorylation in regulating cellular processes, no studies have comprehensively characterized the phosphoproteome in crucian carp. In this study, we report the global phosphoproteome in crucian carp brain and liver during anoxia and reoxygenation. By applying a bottom-up proteomic approach on enriched phosphopeptides we found that the brain phosphoproteome shows surprisingly few changes during anoxia-reoxygenation exposure with only 109 out of 4200 phosphopeptides being differentially changed compared to normoxic controls. By contrast, in the liver 395 out of 1287 phosphopeptides changed. Although most changes occurred in the liver phosphoproteome, the pattern of changes indicated metabolic depression and decreased translation in both brain and liver. We also found changes in phosphoproteins involved in apoptotic regulation and reactive oxygen species handling in both tissues. In the brain, some of the most changed phosphopeptides belonged to proteins involved in central nervous system development and neuronal activity at the synaptic cleft. Changed phosphoproteins specific for liver tissue were related to glucose metabolism, such as glycolytic flux and glycogenolysis. In conclusion, protein phosphorylation in response to anoxia and reoxygenation showed both common and tissue-specific changes related to the functional differences between brain and liver.

## 1 Introduction

The vast majority of animals cannot withstand oxygen depletion for more than a few minutes without experiencing irreversible cell injury and tissue damage. In the absence of oxygen, oxidative phosphorylation is halted, and the energy demand must be covered by the less energy-producing glycolytic pathway (Hochachka and Lutz, 2001). In remarkable contrast, the freshwater fish crucian carp (*Carassius carassius*), can survive without any oxygen (i.e., anoxia) for up to 5 months when temperatures are low (2°C–5°C) (Piironen and Holopainen, 1986). They inhabit small ponds that in the winter are covered by ice and snow, blocking photosynthesis and air diffusion, eventually leaving the water anoxic (Nilsson and Renshaw, 2004; Vornanen et al., 2009). Through a combination of moderate metabolic depression (Nilsson and Lutz, 2004), including a selective reduction of brain activity (Johansson et al., 1995; Nilsson, 2001), and increased glycolysis fueled by a large hepatic glycogen store (Vornanen and Haverinen, 2016), the crucian carp is able to avoid loss of cellular energy charge. Maintained cardiac output (Stecyk et al., 2004) ensures transport of glucose and anaerobic end products. They also possess a unique mechanism where in red muscle an anoxia-activated pathway involving a modified pyruvate dehydrogenase and alcohol dehydrogenase converts lactate to ethanol (Nilsson, 1988; Fagernes et al., 2017). Ethanol is readily excreted over the gills, thereby avoiding cellular lactic acidosis (Nilsson, 1988; Fagernes et al., 2017).

We have previously shown that both proteomic and metabolomic adjustments to anoxia and reoxygenation in the crucian carp are tissue-specific (Dahl et al., 2021; Johansen et al., 2023). Widespread changes of the global proteome did not seem to be the main mechanism for anoxia and reoxygenation adaptations (Johansen et al., 2023), indicating that the metabolomic responses to oxygen concentrations in crucian carp (Dahl et al., 2021) largely result from other processes. While changes in protein levels are relatively slow, as they involve protein synthesis or degradation, phosphorylation/dephosphorylation will provide fast adjustments of protein activity. So far, studies on the regulation of phosphorylation in crucian carp have been limited to key proteins in kinase signaling pathways. The 5′-AMP-activated protein kinase (AMPK) has been suggested to have a key role in initializing metabolic depression in vertebrates, including crucian carp and goldfish (Jibb and Richards, 2008; Stensløkken et al., 2008). One of the main downstream targets of AMPK is the mechanistic target of rapamycin complex 1 (mTORC1), and AMPK-mediated inhibition of mTORC1 reduces energy consuming processes such as protein synthesis (Wullschleger et al., 2006; Rider, 2016). AMPK phosphorylation has been shown to increase during anoxia in crucian carp, but not during severe hypoxia (Stensløkken et al., 2008). In addition, protein kinase B (AKT) phosphorylation and activity decrease in brain and heart during anoxia, potentially having a role in attenuating cell growth and proliferation (Stensløkken et al., 2008).

The most extensive study on phosphorylation in crucian carp so far focused on the mitogen-activated protein kinase (MAPK) pathway, which is another important signaling pathway that regulates cell proliferation and differentiation, apoptosis and cellular responses to external stress (Cowan and Storey, 2003). In crucian carp, phosphorylation of the final effectors of the three MAPK modules, namely, extracellular signal regulated protein kinase (ERK1/2), c-JUN NH_2_ terminal kinase (JNK), and p38-MAPK was regulated in response to anoxia but not hypoxia (Nilsson et al., 2014). Furthermore, the regulation was tissue-specific since phosphorylation of ERK1/2, JNK and p38-MAPK increased in the crucian carp anoxic heart, while in brain only p38-MAPK phosphorylation increased in anoxia (Nilsson et al., 2014). Collectively, the phosphorylation patterns suggested decreasing cell proliferation during anoxia in the investigated tissues (Nilsson et al., 2014). That study did not include liver, thus comparatively little is known about how hepatic phosphorylation is regulated in response to anoxia and reoxygenation.

Adaptation to oxygen depletion has also been extensively studied in anoxia-tolerant freshwater turtles (genera Trachemys and Chrysemys) (Storey, 2007; Bundgaard et al., 2020). However, it is clear that turtles take on a different approach to survival, as turtles enter a deep torpor-like state during anoxia exposure (Lutz and Nilsson, 1997). This contrast in survival strategy is also evident in the AMPK regulation, in which AMPK phosphorylation decreased in the heart and remain unchanged in the liver of the turtle (Rider et al., 2009), while AKT phosphorylation increased in the turtle brain (Nayak et al., 2011).

Previous studies of phosphorylation in anoxia-tolerant vertebrates exposed to anoxia/reoxygenation have been driven by hypotheses generated from studies involving anoxia-intolerant species, and can in that sense be seen as biased towards what is known and steered away from the unknown. They are therefore likely to miss mechanisms that are not expressed in anoxia-sensitive animals and unique to anoxia tolerance. To avoid this bias, we take advantage of the rapidly emerging field of mass spectrometry (MS)-based proteomics, and present the first quantitative study of the global phosphoproteome in crucian carp. We have employed label-free quantitation on brain and liver tissue phosphoproteomes from wild-caught crucian carp exposed in the laboratory to normoxia (control), 5 days of anoxia, and 1 day of reoxygenation with the following goals: 1) to determine whether changes in the phosphoproteome correlate with known physiological adaptations during anoxia-reoxygenation, 2) to investigate to what extent phosphorylation responses are tissue-specific or shared between the two tissues performing very different roles, and 3) to get indications of previously unknown mechanisms regulating responses to anoxia and/or reoxygenation. We focus on brain and liver due to their very different functions and cellular makeup. The brain maintains its activity during anoxia while relying on hepatic glucose supply (Vornanen et al., 2009). The huge glycogen store of the liver seems to be the limiting factor for crucian carp survival during anoxia (Nilsson, 1990).

## 2 Methods

### 2.1 Animal handling

Crucian carp were collected in late summer from the Tjernsrud pond near Oslo, Norway, using nylon net cages. Fish were held at the aquarium facility at the Department of Biosciences, University of Oslo, in tanks with a semi-closed recirculation system supplied with dechlorinated, aerated Oslo tap water in a room with a 12:12 h light:dark cycle. The water temperature varied seasonally, but was kept constant during anoxia exposure, as described below. The fish were fed daily with commercial carp food (Tetrapond, Tetra, Melle, Germany), and acclimated to indoor conditions for at least 4 weeks prior to exposure. All experimental procedures were approved by the Norwegian Animal Health Authority (FOTS ID 16063).

### 2.2 Anoxia exposure and tissue sampling

All fish used in the present study were from the same experimental exposure as previously described (Johansen et al., 2023). We randomly selected crucian carp of both sex (n = 18, weight = 27 ± 8 g) that were transferred to air-tight containers, fasted and acclimated with aerated water (8°C) flowing through the tanks for 48 h prior to anoxia exposure. Anoxia was achieved by continuous low bubbling of nitrogen into the tanks, and the oxygen level monitored using a Firesting fiber-optic oxygen meter with an oxygen probe (PyroScience GmbH, Aachen, Germany). Oxygen levels were maintained at <0.1% of air saturation and the tanks kept anoxic for 5 days. The water temperature was maintained between 8.2°C and 8.6°C throughout the entire experiment. After 5 days of anoxia, nitrogen bubbling was replaced by air bubbling in the tanks, causing the water to become fully air saturated within 1 hour. We collected tissue after 5 days of anoxia; 5 days of normoxia and 5 days anoxia followed by 1 day reoxygenation. Fish were euthanized by a quick blow to the head, the spinal cord was cut, and brain and liver tissues dissected out in the mentioned order. The tissues were snap-frozen in liquid nitrogen and stored at −80°C until further analysis. Crucian carp can withstand at least 2 weeks of anoxic exposure at 8°C (Nilsson, 1990) and we did not observe any mortality during the experiment.

### 2.3 Protein extraction and digestion

Frozen crucian carp brain and liver tissues from 6 fish per experimental group were homogenized with a pestle in ice-cold SILAC™ Phosphoprotein Lysis buffer (Invitrogen, Carlsbad, CA, USA). Homogenized tissue was incubated 5–10 min on ice and then at −80°C, the buffer volume was adjusted to 60 mg tissue/mL lysis buffer and the lysate cleared by centrifugation (18,000 g for 15 min). Total brain protein content was measured with a Detergent Compatible Bradford Assay Reagent (Pierce, Rockford, IL, USA) at 595 nm and total heart protein content was measured with a BCA assay (Pierce, Rockford, IL, USA) at 570 nm, both with BSA as a standard. From each biological replicate, 1 mg protein was precipitated with 5 volumes ice-cold acetone at −20°C overnight. Precipitated protein was pelleted by centrifugation (13,000 g for 15 min), the acetone was aspirated and the pellet let to air dry before resuspension in 6 M urea in 100 mM ammonium bicarbonate. Cystines present in the sample were reduced with 10 mM DTT at 30°C for 60 min and alkylated with 30 mM iodoacetamide at 22°C for 1 hour in the dark. The reaction was quenched with 30 mM DTT at 30°C for 30 min and the sample diluted with 50 mM ammonium bicarbonate before digestion with approximately 1:100 (w/w) trypsin Gold Mass Spec Grade (Promega, Madison, WI, USA) at 37°C overnight. The digest was finally quenched with 1% formic acid and the peptides cleaned by solid-phase extraction (SPE) using a Strata C18-E cartridge (55 μm, 70 Å, Phenomenex, Værlose, Denmark).

### 2.4 Phosphopeptide enrichment

Dried tryptic peptide samples were dissolved in loading buffer (1 M glycolic acid, 6% trifluoroacetic acid, 5% glycerol, and 80% acetonitrile) under continuous shaking. TiO_2_ beads (Titansphere, TiO_2,_ GL Sciences Inc, Japan) were washed in loading buffer 3 times before transferring them to the dissolved tryptic peptide samples. After 1 hour of continuous shaking, the supernatant was collected and transferred to a new tube containing freshly washed TiO_2_ beads for a second incubation. The TiO_2_ beads were collected separately and gently washed sequentially with 200 μL of loading buffer, 200 μL 80% acetonitrile/2% trifluoroacetic acid, 200 mM ammonium glutamate, and 200 μL of 50% acetonitrile/1% trifluoroacetic acid. The TiO_2_ beads were dried and bound peptides were eluted sequentially in 10 min at first with 50 μL of 10% ammonium hydroxide, pH 11.7, then with 50 μL of 15% ammonium hydroxide/60% acetonitrile, and finally with 50 μL of 1% pyrrolidine. Eluted peptides were acidified by adding 75 μL 50% formic acid and cleaned up using ZipTip-C18 (Millipore, Billerica, MA, USA).

### 2.5 LC-MS/MS analysis

The peptide samples were analyzed with an Ultimate 3,000 nano-UHPLC system (Dionex, Sunnyvale, CA, USA) coupled to a Q Exactive mass spectrometer (ThermoElectron, Bremen, Germany) equipped with a nano electrospray ion source. Liquid chromatography (LC) separation was performed with an Acclaim PepMap 100 column (C18, 3 µm beads, 100 Å, 75 μm inner diameter, 50 cm) (Dionex, Sunnyvale CA, USA). We used a flow rate of 300 nL/min with solvent A (0.1% formic acid) and solvent B (0.1% formic acid/90% acetonitrile) and applied a solvent gradient of 4%–35% of solvent B in 160 min, to 50% solvent B in 20 min and finally to 80% of solvent B in 2 min. We operated the mass spectrometer in data-dependent mode to switch automatically between MS and MS/MS acquisition. Survey full scan MS spectra (from m/z 300–2000) were acquired with the resolution R = 70,000 at m/z 200, after accumulation to a target of 1e6. The maximum allowed ion accumulation times were set to 100 m. The used method allowed sequential isolation of up to the 10 most intense ions, depending on signal intensity (intensity threshold 1.7e4), for fragmentation using higher-energy collision induced dissociation (HCD). The target value was 10,000 charges and the resolution set to R = 17,500 with NCE 28. Target ions that were already selected for MS/MS were dynamically excluded for 60 s and the isolation window was m/z = 2 without offset. The maximum allowed ion accumulation for the MS/MS spectrum was 60 m. We enabled the lock mass option in MS mode for internal recalibration during the analysis and accurate mass measurements.

### 2.6 Database search and label-free quantitation

All MS/MS samples were searched against the *Carassius auratus* database (NCBI; all taxons; 96,703 entries), with PEAKS X+ software version 10.5 (Bioinformatics Solutions, Waterloo, ON, Canada). The following parameters were used: digestion enzyme, trypsin; maximum missed cleavage, 1; fragment ion mass error tolerance, 0.1 Da; parent ion error tolerance, 10.0 ppm; and fixed modifications, carbamidomethylation. Oxidation of methionine and acetylation of the N-terminus were specified as variable modifications and the maximum number of PTMs was set to 2. A false-discovery rate of 1% was applied to the datasets. Samples that contained less than 4,200 (brain) or 2000 (liver) peptides were removed, resulting in five (brain) and four (liver) samples from each treatment group that were used for label-free quantitation (LFQ). For LFQ, normalization by total ion count (TIC) was applied, and the following parameters used for filtration: peptide quality ≥2; average area ≥ 1e5; charge between 2 and 5; used peptides ≥1. Phosphorylated peptides passing the applied filters were imported to the Perseus software (Tyanova et al., 2016) for statistical analysis. Peptides had to be identified in at least three replicates in at least one experimental group to be considered (Tyanova and Cox, 2018). Any missing values were replaced by 1e4 before log2 transformation. The overall expression patterns were evaluated by principal component analysis (PCA) using the web tool ClustVis (Metsalu and Vilo, 2015). Data were centered and scaled by unit variance scaling, and singular value decomposition (SVD) with imputation applied to calculate the principal components. Regulation of phosphorylation was assessed on the peptide level by one-way ANOVA and multiple testing corrected for with Tukey’s HSD test applying FDR 5% in Perseus. Phosphopeptides varying by a fold change ≥2 were kept. Hiearchichal clustering was conducted with ClustVis (Metsalu and Vilo, 2015) on normalized and ln(x) transformed data. Rows were centered and scaled by unit variance before clustering using correlation distance and average linkage. Venn diagrams were created using BioVenn (Hulsen et al., 2008). The mass spectrometry proteomics data have been deposited to the ProteomeXchange Consortium via the PRIDE (Perez-Riverol et al., 2022) partner repository with the dataset identifier PXD033061.

### 2.7 Functional enrichment analysis

Goldfish protein sequences of the significantly regulated phosphoproteins were uploaded to BlastKOALA (Kanehisa et al., 2016) to acquire the Uniprot gene names. The entry names were then matched with the corresponding zebrafish names for enrichment analysis. g:Profiler (version e101_e.g.,48_p14_baf17f0) was used for functional profiling with g:SCS multiple testing correction method and a significance threshold of 0.05 (Raudvere et al., 2019). The zebrafish entry list was imported as an unordered query including the entire ANOVA protein list. The KEGG pathway database (Kanehisa, 2019) was used to visualize the relevant enriched pathways.

## 3 Results

### 3.1 Global analysis of brain and liver phosphoproteomes

**FIGURE 1:**
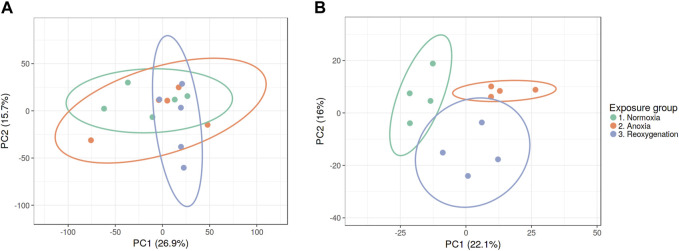
PCA plots of **(A)** brain and **(B)** liver phosphoproteomes during normoxia, anoxia and reoxygenation. Data were normalized and log2 transformed and uploaded to the ClustVis web tool. Unit variance scaling was applied to rows, and SVD with imputation was used to calculate the principal components. Eclipses illustrate 95% confidence intervals.

To explore the impact of oxygen deprivation on the phosphoproteome of crucian carp, we exposed wild-caught crucian carp to 5 days normoxia (air-saturated water), 5 days anoxia (<0.1% of air saturation) and 5 days anoxia followed by 1 day of reoxygenation. Phosphorylated peptides of brain and liver tissues were enriched using TiO_2_ beads and analyzed by LC-MS/MS for label-free quantitation (Supplementary Tables S1, S2). Only phosphopeptides detected in at least three replicates within at least one experimental group were used for further statistical analysis and revealed 4,200 phosphopeptides in the brain and 1,287 phosphopeptides in the liver. Unsupervised PCA (on log2 transformed data) was applied to first examine the global effect on the phosphoproteomes of brain and liver. Interestingly, there was no apparent clustering of experimental groups in the brain phosphoproteome indicating few anoxia-specific changes (Figure 1A). In contrast, the liver phosphoproteome separated into the experimental groups clearly indicating strong effect with anoxia-reoxygenation (Figure 1B).

**FIGURE 2:**
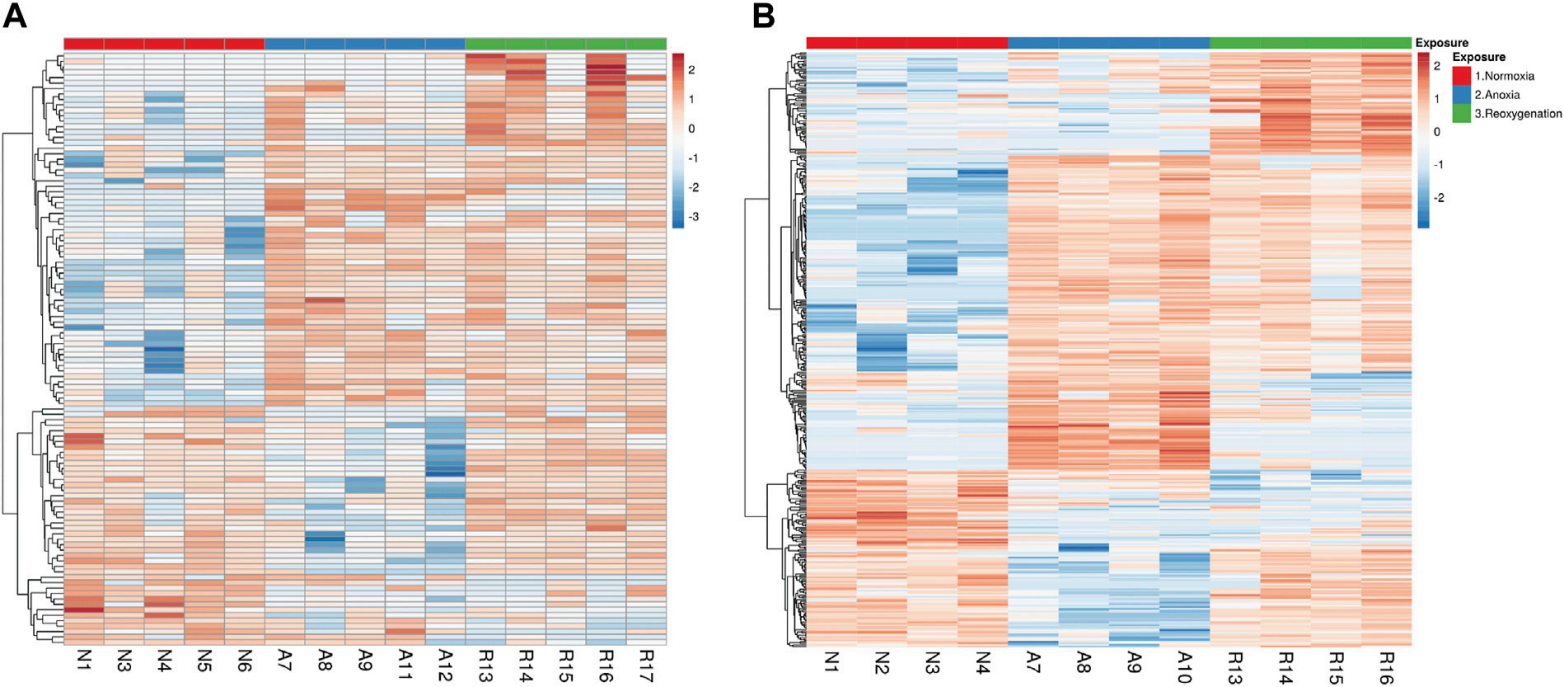
Hierarchal clustering of significantly changed phosphopeptides in the **(A)** brain and **(B)** liver tissue. Phosphopeptides after 5 days normoxia (N), 5 days anoxia **(A)** and 5 days followed by 1 day reoxygenation (R). Input data for heatmaps were ln(x)-transformed, centered and scaled by unit variance. Rows were clustered using correlation distance and average linkage in the ClustVis web tool. The numbering of the samples refers to specific individuals, so that, for example, brain and liver tissue numbered N1 are from normoxic fish number 1.

Next, we applied statistical analysis in combination with filtering out peptides regulated by fold change <2 to focus on the peptides that changed the most in response to anoxia-reoxygenation. The statistical analysis was performed on peptide level rather than protein level since the latter strategy may occult changes of individual peptides within the same protein. Although the total amount of detected phosphopeptides was considerably higher in the brain (4,200 peptides) than in the liver (1,287 peptides), the number of regulated phosphopeptides was much higher in the liver. We found that 109 phosphopeptides in the brain and 395 phosphopeptides in the liver were significantly changed (FDR <5%) compared to normoxia (Figure 2; Supplementary Tables S3, S4). In the brain, 68 phosphopeptides changed in anoxia compared to normoxia (40 increased and 28 decreased in phosphorylation level), and 59 peptides changed in phosphorylation levels in reoxygenation compared to normoxia (49 increased and 10 decreased). The number of phosphopeptides changing in phosphorylation level in anoxia compared to normoxia was 286 in the liver (194 increased and 92 decreased), and 215 liver peptides changed in phosphorylation levels in reoxygenation compared to normoxia (172 increased and 42 decreased). Hierarchically clustering of the significant peptides Figure 2 showed most grouping of peptides according to experimental groups in the liver (Figure 2B).

**FIGURE 3:**
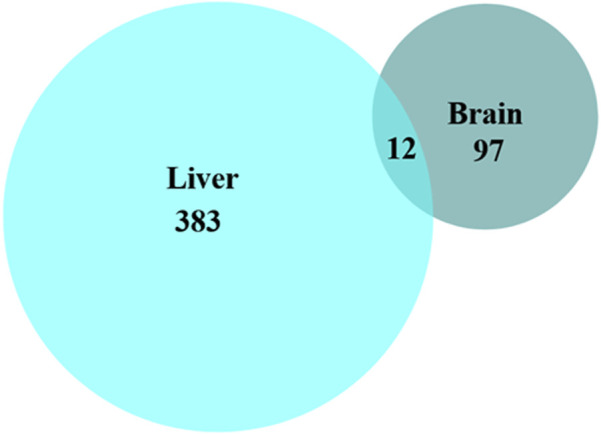
Number of significantly changed phosphopeptides in brain and liver tissue. Protein identities of the shared phosphopeptides (n = 12) present in both brain and liver tissue are shown in Table 1.

Comparison of the changed phosphopeptides revealed that few phosphoproteins were changed at the same phosphosite in both tissues in response to anoxia or reoxygenation (Figure 3). Only 12 phosphopeptides corresponding to 11 phosphoproteins showed similar regulation in both brain and liver (Table 1). Among them, we found proteins involved in kinase signaling (AMPK and MAPK3), apoptotic control (CAST and BADB) and translation initiation (LARP1 and EIF4EBP2), suggesting that these processes are a common response in both tissues to anoxia-reoxygenation conditions in the crucian carp (Table 1).

**TABLE 1:** Regulated phosphoproteins present in both brain and liver tissue. Phosphoproteins with at least one shared and regulated phosphopeptide in both tissues compared to normoxic control. Arrows depict direction (up, upregulated; down, downregulated). Significance threshold was FDR <5%, peptide sequences are shown in Supplementary Tables S3, S4.

			Brain		Liver	
Protein	Gene name	Accession #	Anoxia	Reox	Anoxia	Reox
AMP deaminase 2	AMPD2A	XP_026119538.1		↑	↑	↑
Bcl2-associated agonist of cell death	BADB	XP_026122975.1	↑	↑	↑	↑
Calpastatin-2	CAST	XP_026052327.1	↑	↑	↑	↑
5′-AMP-activated protein kinase subunit beta-1	PRKAB1B	XP_026115112.1	↑	↑	↑	↑
SNW domain-containing protein 1	SNW1	XP_026089561.1	↑		↑	
Thyroid hormone receptor-associated protein 3	THRAP3A	-	↑	↑	↑	
Vacuolar protein sorting-associated protein 13D	VPS13D	XP_026096912.1	↑		↑	
Eukaryotic translation initiation factor 4E-binding protein 2	EIF4EBP2	XP_026134001.1 XP_026117351.1	↓ ↓		↓ ↓	
La-related protein 1	LARP1	XP_026051263.1	↓		↓	
Mitogen-activated protein kinase 1	MAPK3	XP_026064545.1	↓		↓	
Antihemorrhagic factor cHLP-B	AHSG1	XP_026053543.1	↓		↓	↓

### 3.2 Functional enrichment analysis

Differentially changed phosphoproteins (FDR <0.05, fold change >2), including both increased and decreased phosphoproteins, were uploaded to the g:Profiler software for functional enrichment analysis. In response to anoxia-reoxygenation in the brain, enriched gene ontology (GO) terms included L1CAM interactions (Reactome), central nervous system neuron development (biological process) and GTPase binding (molecular function) (Supplementary Table S5). In the liver, the insulin signaling pathway (KEGG pathway) was among the most significantly enriched pathway in response to anoxia-reoxygenation (Supplementary Table S6; Supplementary Figure S1). PKA activation in glucagon signaling (Reactome) and AMPK activity (molecular function) were also enriched in the liver during anoxia-reoxygenation (Supplementary Table S6).

### 3.3 Regulated pathways of interest

Phosphopeptides belonging to AMPK were among the most changing peptides in terms of fold change in both brain and liver (Table 3; Supplementary Tables S3, S4), and AMPK signaling was an enriched GO term in the liver (Supplementary Table S6). Consequently, we investigated in detail the phosphoproteins implicated in the closely related pathways of insulin signaling, PKA activation and AMPK signaling. Because glucose flux is sensitive to insulin and glucagon, and both signaling pathways were enriched in the liver phosphoproteome, we also examined the regulation of glycolytic enzymes in more detail.

#### 3.3.1 AMPK signaling

**FIGURE 4:**
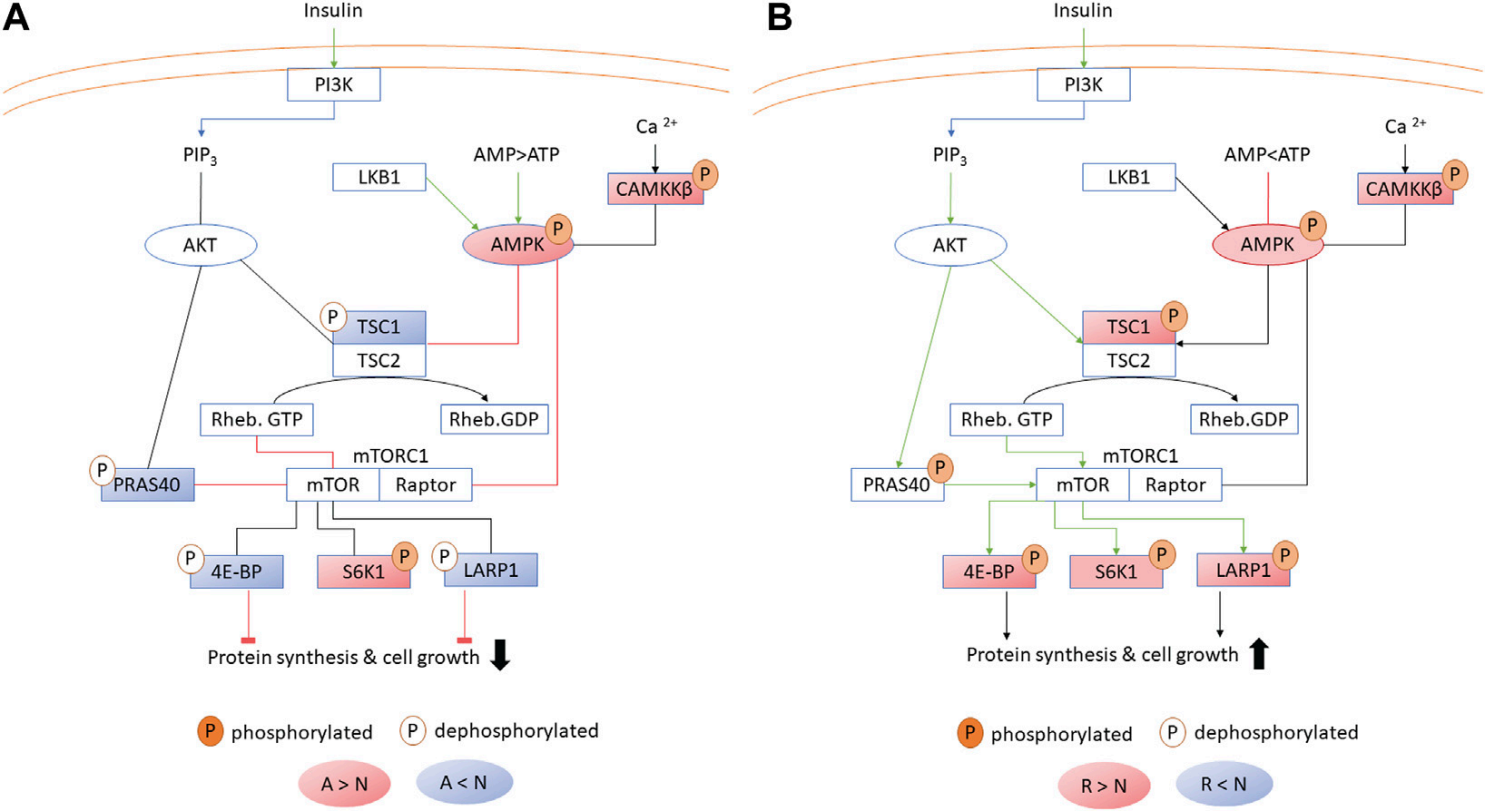
AMPK kinase signaling in the liver during **(A)** anoxia and **(B)** reoxygenation. Significant (FDR <0.05) increase and decrease in phosphorylation compared to normoxia is depicted in red and blue, respectively. The brain experienced similar changes with some exceptions (Table 2). A, anoxia; N, normoxia; R, reoxygenation. Abbreviations otherwise not mentioned in the text: PI3K, phosphoinositide 3-kinase; PIP_3_, phosphatidylinositol (3,4,5)-trisphosphate; Rheb, GTP-binding protein Rheb; LKB1, liver kinase B1.

Phosphoproteins involved in reducing ATP expenditure changed in both brain and liver during anoxia-reoxygenation. Increased phosphorylation of the AMPK subunit beta (PRKAB1B) during anoxia-reoxygenation compared to normoxia occurred at the same residue (corresponding to human Ser-108) in both brain and liver (Figure 4; Tables 2, 3). The specific phosphosite is required for AMPK enzyme activity (Warden et al., 2001) and is located within the carbohydrate binding domain of AMPK (Ovens et al., 2021). Furthermore, we found reduced phosphorylation of Akt1 substrate 1 (PRAS40; proline-rich AKT substrate of 40 kDa), hamartin (TSC1), eukaryotic translation initiation factor 4E-binding proteins (EIF4E-BPs) and La-related protein 1 (LARP1) during anoxia in the liver tissue (Figure 4A; Table 3), and this was also seen for the latter two (EIF4E-BPs and LARP1) in the anoxic brain tissue (Table 2), all compared to normoxia. Although the specific phosphosites have not yet been connected to any specific function, they are in accordance with reduced AKT activity and AMPK-mediated inhibition of mTORC1 activity (Wullschleger et al., 2006). In line with active AMPK, phosphorylation of the MAPK family protein ERK1/2 (MAPK3) decreased in both brain (human Thr-202 and Tyr-204) and liver (Tyr-204) during anoxia compared to normoxia (Tables 2, 3). During reoxygenation, phosphorylation of EIF4E-BPs and LARP1 increased in the liver tissue compared to normoxia (Figure 4B; Table 3), suggesting reactivated mTORC1 and subsequent protein synthesis (Wang and Proud, 2006). In addition, TSC1 phosphorylation increased numerically but not significantly. In the reoxygenated brain, phosphorylation levels of EIF4E-BPs and LARP1 returned to normoxic levels (Table 2).

**TABLE 2:** Phosphopeptides related to AMPK and mTORC1 signaling in the brain. Significantly changed (FDR <5%) phosphopeptides in anoxia and/or reoxygenation compared to normoxia are depicted with arrows. Start-end indicate first and last residue of regulated phosphopeptide (sequence in Supplementary Table S3).

Protein name	Gene name	Accession #	Start-end	Anoxia	Reox
Calcium/calmodulin-dependent protein kinase	CAMK1A	-	353–368		**↓**
	CAMK1A	-	335–342	**↑**	**↑**
Calcium/calmodulin-dependent protein kinase type 1-like	CAMK1A	XP_026118012.1	320–340		**↑**
5′-AMP-activated protein kinase subunit beta-1-like	PRKAB1B	-	106–124	**↑**	**↑**
Eukaryotic translation initiation factor 4E-binding protein 2-like	EIF4EBP2	XP_026134001.1	59–80	**↓**	
	EIF4EBP2	XP_026134001.1	59–80	**↓**	
	EIF4EBP2	XP_026134001.1	60–80	**↓**	
La-related protein 1-like isoform X2	LARP1	XP_026051263.1	659–671	**↓**	
Mitogen-activated protein kinase 1-like	MAPK1	XP_026096459.1	182–200	**↓**	
	MAPK3	XP_026064545.1	204–222	**↓**	

**TABLE 3:** Changed phosphopeptides related to AMPK and mTORC1 signaling in the liver. Significantly changed (FDR <5%) phosphopeptides in anoxia and/or reoxygenation compared to normoxia are depicted with arrows. Start-end indicate first and last residue of regulated phosphopeptide (sequence in Supplementary Table S4).

Protein name	Gene name	Accession #	Start-end	Anoxia	Reox
5′-AMP-activated protein kinase catalytic subunit alpha-1	PRKAA1	XP_026104141.1	334–362	↓	
5′-AMP-activated protein kinase subunit beta-1	PRKAB1A	XP_026129227.1	101–124	↑	↑
	PRKAB1A	XP_026129227.1	106–124	↑	↑
	PRKAB1B	XP_026115112.1	93–116	↑	↑
5′-AMP-activated protein kinase subunit beta-2	PRKAB1A	XP_026132247.1	99–122	↑	↑
	PRKAB1A	XP_026132247.1	104–122	↑	
	PRKAB1A	XP_026132247.1	52–67	↑	
Akt1 substrate 1 (PRAS40)	AKT1S1	XP_026063288.1	128–143	↓	
	AKT1S1	XP_026063288.1	339–348	↓	
Hamartin	TSC1A	XP_026067359.1	368–387	↓	
Eukaryotic translation initiation factor 4E-binding protein 2	EIF4EBP2	XP_026117351.1	17–47	↓	
	EIF4EBP2	XP_026117351.1	59–80	↓	
Eukaryotic translation initiation factor 4E-binding protein 1	EIF4EBP1	XP_026052063.1	19–48	↓	
	EIF4EBP1	XP_026052063.1	61–87	↓	
	EIF4EBP1	XP_026052063.1	61–87	↓	↓
	EIFE3BP3	XP_026105284.1	17–47		↑
La-related protein 1	LARP1	XP_026051263.1	659–671	↓	
La-related protein 1b	LARP1B	XP_026142043.1	765–782		↑
Ribosomal protein S6 kinase alpha-3-like	RPS6KA3A	XP_026112740.1	706–722		↑
Ribosomal protein S6 kinase alpha-4-like	RPS6KA4	XP_026111085.1	718–732	↑	↑
Ribosomal protein S6 kinase beta-1-like	RPS6KB1B	XP_026138368.1	418–432	↑	↑
Mitogen-activated protein kinase 1	MAPK3	XP_026064545.1	204–222	↓	
Calcium/calmodulin-dependent protein kinase kinase 2	CAMKK	XP_026103190.1	532–541	↑	↑
	CAMKK	XP_026103190.1	157–175		↑
cAMP-dependent protein kinase catalytic subunit alpha-like isoform X1	PRKACAA	XP_026074776.1	350–372	↑	↑
	PRKACAA	XP_026074776.1	18–46		↑
cAMP-dependent protein kinase	PRKACBB	-	199–217	↑	
cAMP-dependent protein kinase type II-alpha regulatory subunit-like	PRKAR2AA	XP_026126607.1	96–116	↑	↑
Insulin receptor substrate 2-B-like	IRS2B	XP_026123814.1	355–371	↓	

Calcium/calmodulin-dependent protein kinase kinase 2 (CAMKK2) phosphorylation at Ser-495 (human sequence) increased in the liver during anoxia and reoxygenation compared to normoxia (Figure 4; Table 3). Phosphorylation at this site deactivates CAMKK2, and has been shown to be phosphorylated by cAMP-dependent protein kinase (PKA; PRKACC) (Langendorf et al., 2020). PKA phosphorylation at Thr-197 (human sequence), required for its activity (Cauthron et al., 1998), increased in anoxia and non-significantly in reoxygenation compared to normoxia in the liver (Table 3). CAMKK2 phosphorylation did not change in the brain, while three phosphosites in calcium/calmodulin-dependent protein kinase (CAMK1) increased in phosphorylation during anoxia and reoxygenation compared to the normoxic controls (Table 2). Also phosphorylation of three different isoforms of ribosomal protein (p70) S6 kinase (RPS6K, S6K1) increased in the liver during anoxia and reoxygenation compared to normoxia (Figure 4; Table 3). Phosphorylation at one of these phosphosites, Ser-421 (corresponding to Ser-424 in human sequence), has been reported to be involved in the activation of RPS6K (Zhang et al., 2013). Phosphorylation of the insulin receptor substrate 2 (IRS2B) decreased during anoxia compared to normoxia in the liver at Ser-357 (Table 3; Supplementary Figure S1) which corresponds to Ser-270 and Ser-306 in human IRS 1 and 2, respectively. Phosphorylation at this site has been shown to inhibit downstream insulin signaling (Zhang et al., 2008; Shaw, 2011).

#### 3.3.2 Glucose metabolism

**FIGURE 5:**
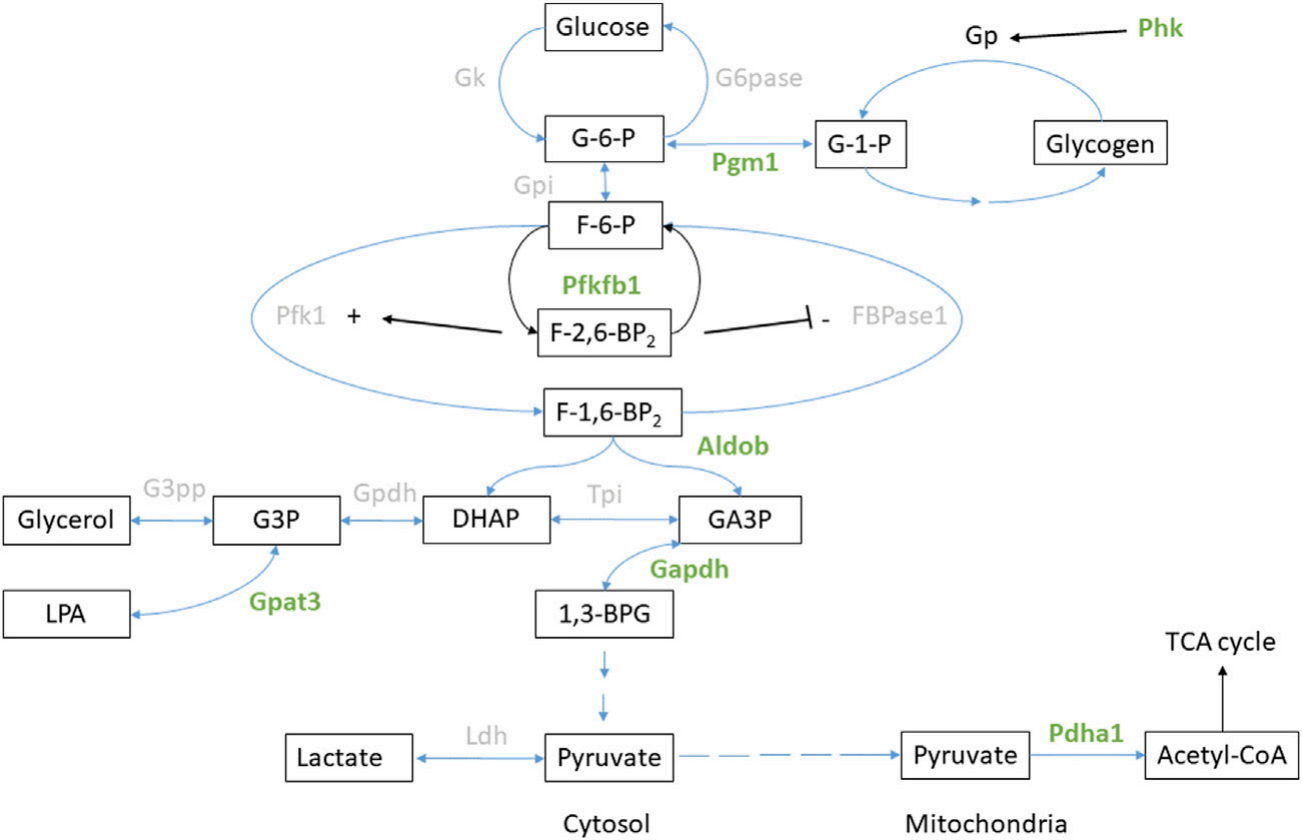
Glucose metabolism in the liver. Enzymes with significant regulation (FDR <0.05) of phosphorylation during anoxia-reoxygenation are highlighted in green. Details are found in Table 3. Abbreviations not mentioned in the text: GK, glucokinase; G6Pase, glucose-6-phosphatase; GP, glycogen phosphorylase; GPI, glucose-phosphate isomerase; FBPase1, Fructose 2,6-bisphosphatase; TPI, triosephosphate isomerase, GPDH, glycerol-3-phosphate dehydrogenase; G3PP, glycerol-3-phosphate phosphatase; LPA, lysophosphatidic acid; LDH, lactate dehydrogenase.

Changing phosphorylation patterns of proteins related to glucose metabolism were especially apparent in the liver tissue (Figure 5). Glycogen catabolism is regulated through phosphorylation of glycogen phosphorylase by phosphorylase b kinase (Johnson, 1992). In the liver, we found increased phosphorylation levels of phosphorylase b kinase alpha (PHKA2) at Ser-729, Ser-980 and Ser-984 during anoxia and at Ser-984 during reoxygenation, while Ser-969 and Thr-979 decreased during anoxia (Table 4), all compared to normoxic controls. In subunit beta (PHKB), phosphorylation increased at position Ser-701 in the reoxygenated liver compared to normoxia (Table 4).

**TABLE 4:** Changed phosphopeptides related to glucose metabolism in the liver. Significantly changed (FDR <5%) phosphopeptides in anoxia and/or reoxygenation compared to normoxia are depicted with arrows. Start-end indicate first and last residue of regulated phosphopeptide (sequence in Supplementary Table S4).

Protein name	Gene name	Accession #	Start-end	Anoxia	Reox
Phosphorylase b kinase alpha	PHKA2	XP_026077329.1	719–735	↑	
	PHKA2	XP_026070865.1	972–993	↑	↑
	PHKA2	XP_026070865.1	969–993	↓	
Phosphorylase b kinase beta	PHKB	XP_026071435.1	692–716		↑
6-phosphofructo-2-kinase/fructose-2,6-bisphosphatase	PFKFB1	XP_026097313.1	27–49	↑	↑
	PFKFB1	XP_026099603.1	40–62	↑	
Phosphoglucomutase-1	PGM1	XP_026069410.1	106–130	↓	
	PGM1	XP_026069410.1	108–130	↓	
	PGM1	XP_026120783.1	108–130		↑
Fructose-bisphosphate aldolase B	ALDOB	XP_026098543.1	260–297	↑	
	ALDOB	XP_026098543.1	216–243		↓
Glyceraldehyde-3-phosphate dehydrogenase	GAPDH	XP_026084801.1	161–184	↑	↑
	GAPDH	XP_026084801.1	161–192	↑	↑
	GAPDH	XP_026140054.1	161–184	↑	
	GAPDH	XP_026140054.1	161–192	↑	
Glycerol-3-phosphate acyltransferase 3 (GPAT3)	AGPAT9L	XP_026101554.1	109–122	↑	↑
	AGPAT9L	XP_026096261.1	110–127	↑	
Pyruvate dehydrogenase E1 component subunit alpha	PDHA1A	XP_026112693.1	299–312	↓	↓
	PDHA1A	XP_026112693.1	299–312	↓	↓
	PDHA1A	XP_026112693.1	299–312	↓	↓
	PDHA1A	XP_026066414.1	292–305	↓	↓
	PDHA1A	XP_026066414.1	292–305	↓	↓

Related to glycolytic flux direction, we observed differential phosphorylation of 6-phosphofructo-2-kinase/fructose-2 6-bisphosphatase (PFKFB1) in the liver during anoxia. PFKFB1 regulates the concentration of fructose-2,6-bisphosphate, an important allosteric factor for phosphofructokinase (PFK1) and thus a stimulator of glycolysis (Rider et al., 2004). PFKFB-1 phosphorylation at position Ser-33 (corresponding to human sequence) increased during anoxia and reoxygenation compared to normoxia in the liver (Table 4). Phosphorylation at Ser-33 has been reported to stimulate the phosphatase activity of the protein, thus facilitating active gluconeogenesis (Kurland and Pilkis, 1995).

Additionally, hepatic phosphoglucomutase 1 (PGM1) phosphorylation decreased at Thr-115 and Ser-117 during anoxia compared to normoxia (Table 4). Hepatic fructose-bisphosphate aldolase b (ALDOB) phosphorylation levels increased at Ser-272 in anoxia and decreased at Thr-241 in reoxygenation while phosphorylation levels of Thr-182 increased in hepatic glyceraldehyde-3-phosphate dehydrogenase (GAPDH) during anoxia-reoxygenation, all compared to normoxic controls (Table 4). Glyceraldehyde-3-phosphate acyltransferase (GPAT3) phosphorylation at Ser-111 increased in liver during anoxia and reoxygenation compared to normoxia (Table 4). Pyruvate dehydrogenase E1 subunit alpha (PDHA1A) phosphorylation decreased in both anoxia-reoxygenation compared to the normoxic control at position Ser-293 in the liver (Table 4).

## 4 Discussion

A lack of oxygen is fatal for most vertebrates within minutes, but for the anoxia-tolerant crucian carp anoxic periods often occur each winter in their natural habitat. Such long-term anoxia requires extraordinary measures to survive, and not surprisingly, the crucian carp exhibits crucial and unique adaptations. In this study, we have quantified the global phosphoproteomes of crucian carp brain and liver by label-free quantitation of LC-MS/MS data to explore how protein phosphorylation levels are affected by anoxia and reoxygenation in this anoxia-tolerant species. Since the proteome of crucian carp exhibits relatively few (but important) changes in response to anoxia and reoxygenation (Johansen et al., 2023), and since post-translational modifications can respond fast to external cues and internal signaling, we expected a relatively large number of changes in the phosphorylation status of proteins in anoxic and reoxygenated brain and liver. Further, we predicted that even though the crucian carp maintains a moderate level of neuronal activity during oxygen depletion (Johansson et al., 1995), many processes that are oxygen related would also show changes in the phosphorylation pattern. However, our data indicated that the brain phosphoproteome was only moderately regulated by the extreme changes in oxygen concentrations compared to that of the liver. From the PCA plots of the total phosphoproteome, we observed no clustering of brain phosphopeptides throughout the anoxia-reoxygenation exposure (Figure 1A), indicating that no major global phosphoproteomic regulation occurred in response to anoxia-reoxygenation. Most brain tissue adaptations to anoxia and reoxygenation thus seem to be regulated by mechanisms that do not heavily rely on phosphorylation, or at least only subtle changes of some few key proteins. The levels of 109 phosphopeptides corresponding to 92 proteins did significantly change in the brain during anoxia-reoxygenation, and of course, many of these changes may serve important functions. Few GO terms were enriched in the brain phosphoproteome, but the few that were significantly enriched were involved in nervous system development and presynaptic activity (Supplementary Table S5). The specific phosphorylation sites of these proteins are not well-described in the literature to date, but they do suggest that the selective and moderate suppression of brain activity in anoxic crucian carp (Nilsson and Lutz, 2004) could be partially regulated through phosphorylation. This study was performed on the whole brain phosphoproteome. Because brain tissue is quite heterogeneous, with many cell types and regions performing different tasks, local changes in phosphorylation state are likely and may not be detected at the whole brain level. The study will therefore primarily have detected changes that are of a general nature and widespread in the brain tissue.

In contrast to the brain, the liver phosphoproteome samples clearly clustered according to the experimental groups in both the PCA plot (Figure 1B) and the heatmap (Figure 2B). The number of regulated phosphopeptides was 395 in liver, corresponding to 253 proteins. The liver tissue in fasting crucian carp is engaged in oxidative degradation of lipids and proteins during normoxia, while in anoxia glycogenolysis dominates (Lutz and Nilsson, 1997), illustrating the substantial change in use of energy sources depending on oxygen availability. This was indeed illustrated by the enrichment of GO terms related to energy metabolism in the liver during anoxia-reoxygenation both at the proteome level (Johansen et al., 2023) and the phosphoproteome level (Supplementary Table S6). The combination of metabolic suppression to reduce ATP demand and the increased hepatic glucose export to supply glycolytic ATP production are, as previously mentioned, key mechanisms for long-term anoxic survival in the crucian carp (Vornanen et al., 2009). We were therefore particularly interested in the changes of phosphorylation in pathways related to energy metabolism. In the following, we will also comment on the potential impact of changed levels of phosphorylation implicated in cell survival and handling of reactive oxygen species (ROS), since several phosphoproteins involved in these processes displayed large fold changes in phosphorylation (Supplementary Tables S3, S4).

### 4.1 Energy conservation in anoxia

AMPK is a well-known key regulator of metabolic depression (Hardie and Ashford, 2014) and was differentially phosphorylated in this study. The observed increase in AMPK phosphorylation in anoxic brain and liver stimulates AMPK activity, and are in line with previously observed AMPK alpha phosphorylation of the activating Thr-172 in anoxic crucian carp brain, heart and liver (Stensløkken et al., 2008) and hypoxic goldfish liver (Jibb and Richards, 2008). AMPK phosphorylation was still high after 1 day of reoxygenation in brain and liver (Figure 4), consistent with the maintained high phosphorylation levels of AMPK in crucian carp brain after 7 days of reoxygenation (Stensløkken et al., 2008). The phosphorylation status of AMPK suggests maintained activity throughout reoxygenation. However, we have previously found that within 3 hours of reoxygenation the hepatic ATP level even overshoots the normoxic level and that the AMP level has fully recovered (Dahl et al., 2021). The resultant high ATP:AMP ratio should mean that ATP outcompetes AMP for binding to AMPK, inactivating the enzyme regardless of phosphorylation status (Li et al., 2017). Taken together with the phosphorylation pattern of downstream AMPK targets during reoxygenation (Figure 4), this suggests that AMPK, even if still phosphorylated, is not active during reoxygenation in the crucian carp.

mTORC1 has recently gained attention due to its key role in metabolic stress responses in hypoxia-tolerant animals (Wu and Storey, 2021). In anoxia-intolerant mammals, oxygen depletion triggers AMPK activation, which in turn phosphorylates the TSC1/2 complex and inhibits mTORC1 activity (Wullschleger et al., 2006). In contrast, hypoxia-tolerant naked mole rats (*Heterocephalus glaber*) and red-eared slider turtles (*Trachemys scripta elegans*) experience increasing mTORC1 activities in response to hypoxia and anoxia, respectively (Szereszewski and Storey, 2018; Wu and Storey, 2021). In the case of crucian carp, AMPK is activated only in response to anoxia, and not hypoxia (Stensløkken et al., 2008). Even though we did not detect mTORC1 phosphorylation in this present study, our findings of reduced phosphorylation of mTORC1-associated proteins (PRAS40, TSC1) and downstream targets (EIF4E-BPs, LARP1) collectively suggest that mTORC1 activity in the crucian carp liver is reduced (Figure 4). It seems likely that this inhibition is a result of AMPK activation.

Protein synthesis in crucian carp has been shown to be relatively unaffected but possibly slightly suppressed in brain, but heavily inhibited (>95%) in liver during anoxia (Smith et al., 1996). Our results suggest that this stall in translation could be regulated through phosphorylation of translation initiation binding proteins (Table 1). The majority of the identified EIF4E-BP phosphopeptides returned to normoxic phosphorylation levels after 1 day of reoxygenation (Table 3) in line with the reported normoxic translational rates in crucian carp liver after 24 hours reoxygenation (Smith et al., 1996). In addition, dephosphorylation of LARP1 has been attributed to inhibiting translation (Fonseca et al., 2015), suggesting that the observed reduction in LARP1 phosphorylation in anoxic crucian carp liver (Figure 4A) also participates in suppressing translation. Our data therefore suggest that brain protein synthesis is reduced to some extent, since EIF4E-BP and LARP1 phosphorylation decreased in the anoxic brain (Table 2).

### 4.2 Glucose metabolism

Glucose supplied by from a huge hepatic glycogen store is the main energy source for most crucian carp tissues during anoxia and crucial for long-term survival (Vornanen and Haverinen, 2016). In response to anoxia-reoxygenation, we found changes in levels of phosphoproteins involved in hepatic glycogen metabolism (PHKA2, PGM1) and glucose flux in glycolysis/gluconeogenesis (PFKFB1, GPAT3). In the liver, phosphorylation of PHKA2 (alpha subunit of phosphorylase b kinase) increased during anoxia (Table 4). Interestingly, phosphorylation of PHKB (beta subunit of phosphorylase b kinase) at Ser-684 increased in the anoxic brain (Supplementary Table S3), in contrast to the reduced phosphorylation levels at Ser-701 in the liver (Table 4). The fact that this was the only glucose-related enzyme that appeared to be regulated by phosphorylation in the brain is interesting and probably reflects that crucian carp brain is well protected from energy depletion during anoxia.

The observed increase in phosphorylation of PFKFB-1 in the liver indicates a possible degradation of fructose-2,6-bisphosphate during anoxia and reoxygenation, and is probably a result of glucagon-mediated PKA phosphorylation (Kurland and Pilkis, 1995; Janah et al., 2019). Fructose-2,6-bisphosphate has been shown to be the most important allosteric factor for PFK1 activity (Uyeda et al., 1981), and reduced concentrations are coupled to attenuated glycolysis and stimulated gluconeogenesis (Uyeda et al., 1981; Ros and Schulze, 2013). While this makes sense during reoxygenation, when liver tissue starts to rebuild the glycogen stores (Vornanen and Haverinen, 2016), it was more surprising to find the same PFKFB-1 phosphorylation pattern also in anoxia. However, reduced concentrations of fructose-2,6-bisphosphate have previously been observed in anoxic goldfish liver (Storey, 1987), supporting degradation of this allosteric factor. Taken together, we hypothesize that simultaneous glycolysis and gluconeogenesis is occurring in hepatic tissue during anoxia-reoxygenation (Moreno et al., 2014).

### 4.3 Cell survival and ROS

One of the proteins that exhibited high fold changes in both brain and liver during anoxia exposure and recovery was the BCL2-associated agonist of cell death (BADB). Phosphorylation at Ser-118 of BADB increased during anoxia and reoxygenation in both tissues (Table 1) and has been reported to subsequently inactivate the protein by binding to 14-3-3 proteins, thereby hindering apoptosis and stimulating cell survival (Downward, 1999; Datta et al., 2000). This finding is in line with the increased gene expression of the anti-apoptotic proteins BCL-2 and BCL-XL during anoxia in the anoxia-tolerant turtle (Krivoruchko and Storey, 2015). Furthermore, BCL-2 knockdown in turtle neuronally enriched primary cell cultures caused increased apoptosis and ROS levels during anoxia-reoxygenation (Kesaraju et al., 2014). A moderate increase in cell death after reoxygenation has been observed in crucian carp brain, but not coinciding with changes in caspase-3 transcript expression (Lefevre et al., 2017). Part of the caspase-3 activation occurs through BCL-2-mediated cytochrome c release and downstream activation of caspase-3 (Birkinshaw and Czabotar, 2017). Similarly as BCL-2 suppression in turtle activates caspase-3 (Kesaraju et al., 2014), phosphorylation and inactivation of BADB would contribute to the inhibition of this pathway, and could explain why the increased neuronal cell death is probably not linked to caspase-3. Other proteins involved in apoptotic control did also change in phosphorylation levels during anoxia-reoxygenation in the brain and the liver. For example, apoptotic chromatin condensation inducer in the nucleus (ACIN1) exhibited one of the most elevated phosphorylation levels in the anoxic liver compared to normoxia (Supplementary Table S4). Furthermore, programmed cell death protein 4 (PDCD4A), calpastatin (CAST), translationally-controlled tumor protein homolog (TPT1), forkhead box protein O1-A (FOXO1A) and 14-3-3- gamma protein (YWHAH) all underwent changing phosphorylation patterns in the liver (Supplementary Table S4), while PDCD4B and CAST phosphorylation were also regulated in the brain (Supplementary Table S3). Although the precise functions of these phosphorylation sites are scarcely described in the literature, they still point towards broad regulation of apoptosis in the brain and liver during anoxia and reoxygenation.

Interestingly, among the phosphopeptides that exhibited the highest fold changes in the liver during anoxia-reoxygenation we found the ROS scavenging enzymes superoxide dismutase [Cu-Zn] 1 (SOD1) and peroxiredoxin (PRDX; Supplementary Table S4). However, no functionality has so far been reported for the regulated phosphosites in either SOD1 or PRDX. In the goldfish, total SOD activity (including both cytoplasmic SOD1 and mitochondrial SOD2) was unchanged in the brain and liver during anoxia and reoxygenation (Lushchak et al., 2001). In the moderately anoxia-tolerant salamander, SOD activity did remarkably decrease during reoxygenation (Issartel et al., 2009), while antioxidant activities decreased during anoxia in anoxia-tolerant turtles (Willmore and Storey, 1997). Clearly more than one strategy for ROS handling during oxygen depletion and recovery exist, and the exact mechanisms occurring in the crucian carp remain to be elucidated.

### 4.4 Conclusion

Although a substantial higher number of phosphopeptides were identified in the brain phosphoproteome compared to the liver phosphoproteome, the majority of differentially changed phosphopeptides were found in the liver. Some of the phosphopeptides showing the biggest change in brain belonged to proteins involved in neuronal activity at the synaptic cleft. Although no functionality could be assessed to the phosphosites in this study, their differentiation still indicates a role in synaptic activity that calls for further studies. In the liver, changed phosphoproteins were related to glycolytic flux and glycogenolysis, Common in both tissues, regulation towards metabolic depression through AMPK-signaling was apparent. Moreover, our data show strong regulation of phosphosites in proteins involved in cell death regulation and ROS handling and indicate a direction towards cell survival rather than apoptosis in response to oxygen depletion and recovery. This could presumably be made possible through inhibition of apoptotic pathways and low ROS accumulation, either due to low ROS generation or high antioxidant activity. More focus should be given to the functional impact of these processes during anoxia and reoxygenation. This work also emphasizes the need for including the understudied and important liver tissue more systematically in future studies to elucidate how the crucian carp survive anoxia-reoxygenation.

## Data Availability

The datasets presented in this study can be found in online repositories. The names of the repository/repositories and accession number(s) can be found below: http://www.proteomexchange.org/, PXD033061.
