# Supplementary material for: Phosphoproteomic changes in response to anoxia are tissue-specific in the anoxia-tolerant crucian carp (Carassius carassius)
Source: Front Physiol. 2024 May 30;15:1407834. doi: 10.3389/fphys.2024.1407834 (PMC11170284; doi:10.3389/fphys.2024.1407834)
Supplement: Supplementary file 2 [file DataSheet1.docx]

Supplementary Material

## Supplementary Figures


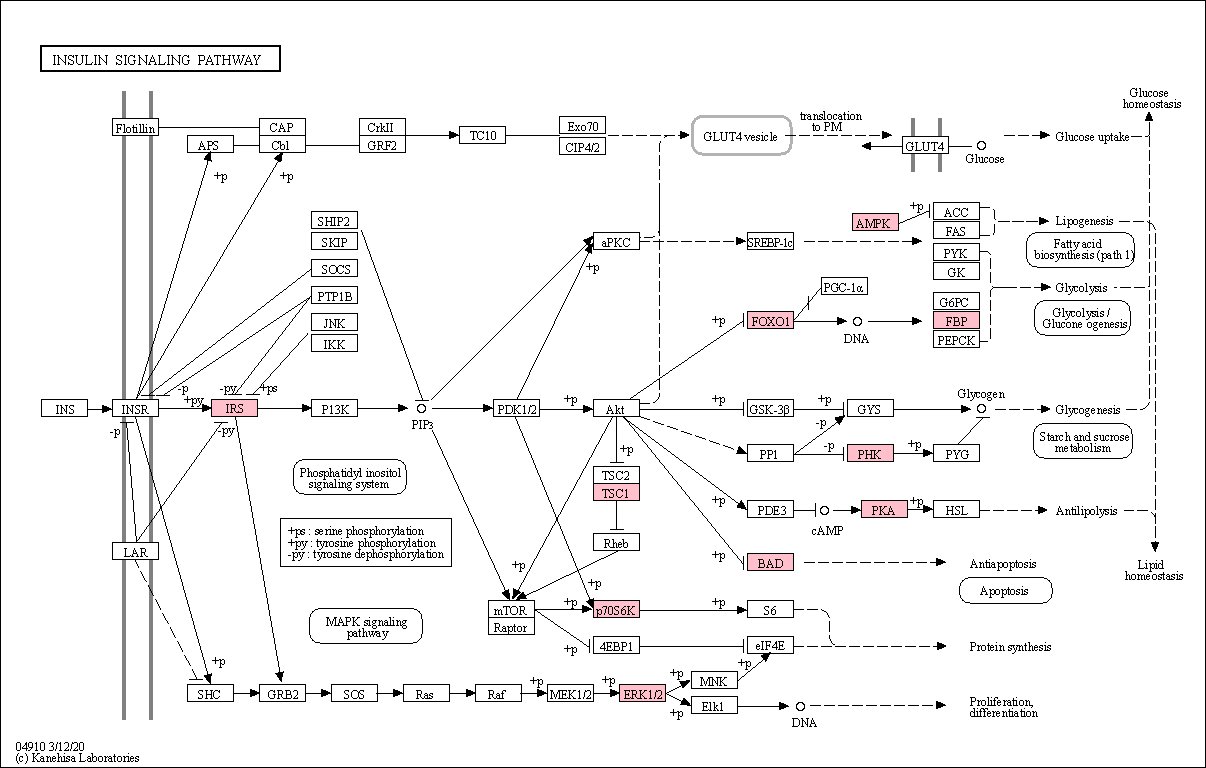


**Supplementary Figure 1.** Insulin signaling pathway (KEGG pathway based on *Danio rerio).* Liver proteins with regulated phosphopeptides in anoxia and/or reoxygenation compared to normoxia are colored in pink (as detected by KEGG).
